# Total Flavonoids of Rhizoma Drynariae Ameliorate Rheumatoid Arthritis by Modulating Macrophage Polarization and HIF-1α-Associated Glycolysis

**DOI:** 10.3390/ph19101592

**Published:** 2026-10-08

**Authors:** Zhenya Liu, Silu Li, Wenjia Zhao, Jiayuan Li, Yujie Yang, Hengjun Huang, Chengyu Yang

**Affiliations:** 1 Jiangxi Province Key Laboratory of Traditional Chinese Medicine Pharmacology, Institute of Traditional Chinese Medicine Health Industry, China Academy of Chinese Medical Sciences, Nanchang 330115, China; 18864855859@163.com (Z.L.); lisilu199811@163.com (S.L.); 18770910458@163.com (W.Z.); jyl@itcmhi.ac.cn (J.L.); yyj990519@163.com (Y.Y.); 2 Institute of Chinese Materia Medica, China Academy of Chinese Medical Sciences, Beijing 100700, China; 3 Jiangxi Health Industry Institute of Traditional Chinese Medicine, Nanchang 330115, China

**Keywords:** total flavonoids of Rhizoma Drynariae, rheumatoid arthritis, glycolysis, macrophage polarization, HIF-1α

## Abstract

**Objective:** Rheumatoid arthritis (RA) is a chronic autoimmune disease with the abnormal accumulation of pro-inflammatory macrophages within the synovial joints. Total flavonoids of Rhizoma Drynariae (TFRD), the major bioactive constituents of the traditional Chinese medical herb *Drynariae rhizome*, have shown potent activities in treating RA, but their effect and underlying mechanism in modulating macrophage behavior remain unknown. This study aimed to evaluate the therapeutic potential of TFRD in regulating macrophage polarization during RA, and to further elucidate the underlying mechanism. **Methods:** The therapeutic effects of TFRD were assessed by utilizing a collagen-induced arthritis (CIA) mice model in vivo. A macrophage polarization model was employed to investigate the effect of TFRD on the macrophage pro-inflammatory polarization and glycolytic activity in vitro. Additionally, the role of hypoxia-inducible factor-1α (HIF-1α) in the TFRD-mediated regulation of macrophage glycolysis was examined via plasma transfection. **Results:** Administration of TFRD significantly reduced the arthritis severity scores and ameliorated joint swelling and bone destruction in CIA mice. The therapeutic efficacy of high-dose TFRD (TFRD-H) was broadly similar to that of MTX in CIA mice. Further, TFRD decreased the proportion of pro-inflammatory macrophages and the expression of HIF-1α within the joints of CIA mice. The in vitro experiments indicated that TFRD inhibited the lipopolysaccharide-induced macrophage pro-inflammatory polarization. Mechanistically, TFRD attenuated glycolytic metabolism and downregulated HIF-1α expression during pro-inflammatory polarization, while HIF-1α overexpression partly reversed these effects. Furthermore, 16S rRNA sequencing analysis revealed the mitigated dysbiosis of the gut microbiota and increased relative abundance of Parabacteroides in CIA mice. **Conclusions:** These findings indicate that TFRD alleviated RA, which was partly dependent on downregulating pro-inflammatory macrophages and mitigating HIF-1α-associated glycolysis. This study could provide a scientific basis for further research and the clinical use of TFRD.

## 1. Introduction

Rheumatoid arthritis (RA) is a systemic autoimmune disorder characterized by symmetrical polyarthritis, synovial hyperplasia and progressive joint destruction, ultimately resulting in functional impairment and disability in patients [1]. Epidemiological data indicate that the global prevalence of RA was approximately 0.5% to 1% in 2020 and could reach 31.7 million cases in 2050 [2,3]. Currently, non-steroidal anti-inflammatory drugs and glucocorticoids constitute the primary pharmacological treatments for RA [4]. However, these strategies mainly provide symptomatic relief and slow disease progression, and they are often accompanied by adverse reactions, including leukopenia and hepatorenal damage [4]. Consequently, the development of safer and more efficacious therapeutic strategies remains a critical clinical priority.

Macrophages are highly diverse phagocytes that have been shown to play important roles in autoimmune diseases [5]. In response to various stimuli, naive macrophages could differentiate into either pro-inflammatory or anti-inflammatory phagocytes. During RA progression, the population of pro-inflammatory macrophages is abnormally elevated in both peripheral blood and synovial tissue [6,7], and they secrete large amounts of TNF-α, IL-6, and IL-1β, thereby amplifying synovitis and activating osteoclasts [8]. Metabolic reprogramming plays a critical role during macrophage polarization, with pro-inflammatory macrophages characterized by elevated glycolysis and pentose phosphate pathway (PPP) activity, coupled with impaired tricarboxylic acid (TCA) cycle flux [9]. Moreover, emerging evidence indicates that inhibition of glycolysis could trigger the phenotypic transition of pro-inflammatory macrophages to an anti-inflammatory state [10,11,12]. Indeed, mitigating glycolysis could drive pro-inflammatory-to-anti-inflammatory phenotypic switching and ameliorate synovial inflammation in animal models of RA [12]. Therefore, efficient modulation of glycolysis represents a potential therapeutic strategy for RA.

Hypoxia-inducible factor-1α (HIF-1α), a key transcription factor essential for cellular adaptation to low-oxygen conditions, can influence macrophage polarization through the regulation of glycolysis [13,14]. Under hypoxic or inflammatory conditions, HIF-1α translocates into the nucleus and activates the expression of GLUT1 and HK2, the glycolytic rate-limiting enzymes, thereby enhancing glycolytic metabolism and promoting pro-inflammatory polarization [15,16]. Meanwhile, glycolytic intermediates could further stabilize HIF-1α, forming a positive-feedback loop that sustains the pro-inflammatory phenotype of macrophages [17]. In addition, elevated HIF-1α expression has been observed in the synovia and sera of patients with RA and has been linked to synovial angiogenesis [18,19,20]. Recent evidence indicates that RA serum induces macrophage pro-inflammatory polarization via activation of the glycolysis–HIF-1α axis [21], suggesting that HIF-1α may serve as a potential target for regulating the pro-inflammatory/anti-inflammatory balance in RA.

*Drynariae rhizoma* (Gusuibu), derived from the dried rhizome of *Drynaria fortunei* J. Sm., has been traditionally utilized in Chinese medicine to treat fractures, musculoskeletal pain, and various orthopedic disorders. The total flavonoids of Rhizoma Drynariae (TFRD), the major bioactive constituents of *Drynariae rhizoma*, possess diverse pharmacological activities and are clinically applied in the management of osteoporosis [22,23]. Recent studies have reported the therapeutic potential of TFRD against RA in animal models and investigated the involvement of Th17 cell differentiation in this process [24,25]. However, whether TFRD regulates macrophage polarization in RA has not been reported.

Here, we utilized a collagen-induced arthritis (CIA) mouse model to systematically evaluate the anti-arthritic effect of TFRD. We found that TFRD alleviated joint damage, reduced the population of pro-inflammatory macrophages and restored the gut microbiota composition in CIA mice. Moreover, we explored the involvement of the HIF-1α-dependent glycolytic metabolism TFRD-mediated inhibition of macrophage pro-inflammatory polarization. This study aims to provide new pharmacological insights into the mechanism of TFRD in the treatment of RA, and to support its further development as a potential therapeutic agent.

## 2. Results

### 2.1. TFRD Alleviate Arthritis in CIA Mice

To control the quality of TFRD, the UPLC-MS analysis was performed to analyze a standard sample (containing naringin and neoeriocitrin) and TFRD power. As shown in Figures S1 and S2, naringin and neoeriocitrin, both recognized as main bioactive constituents of TFRD [25], were identified in TFRD power.

**Figure 1:**
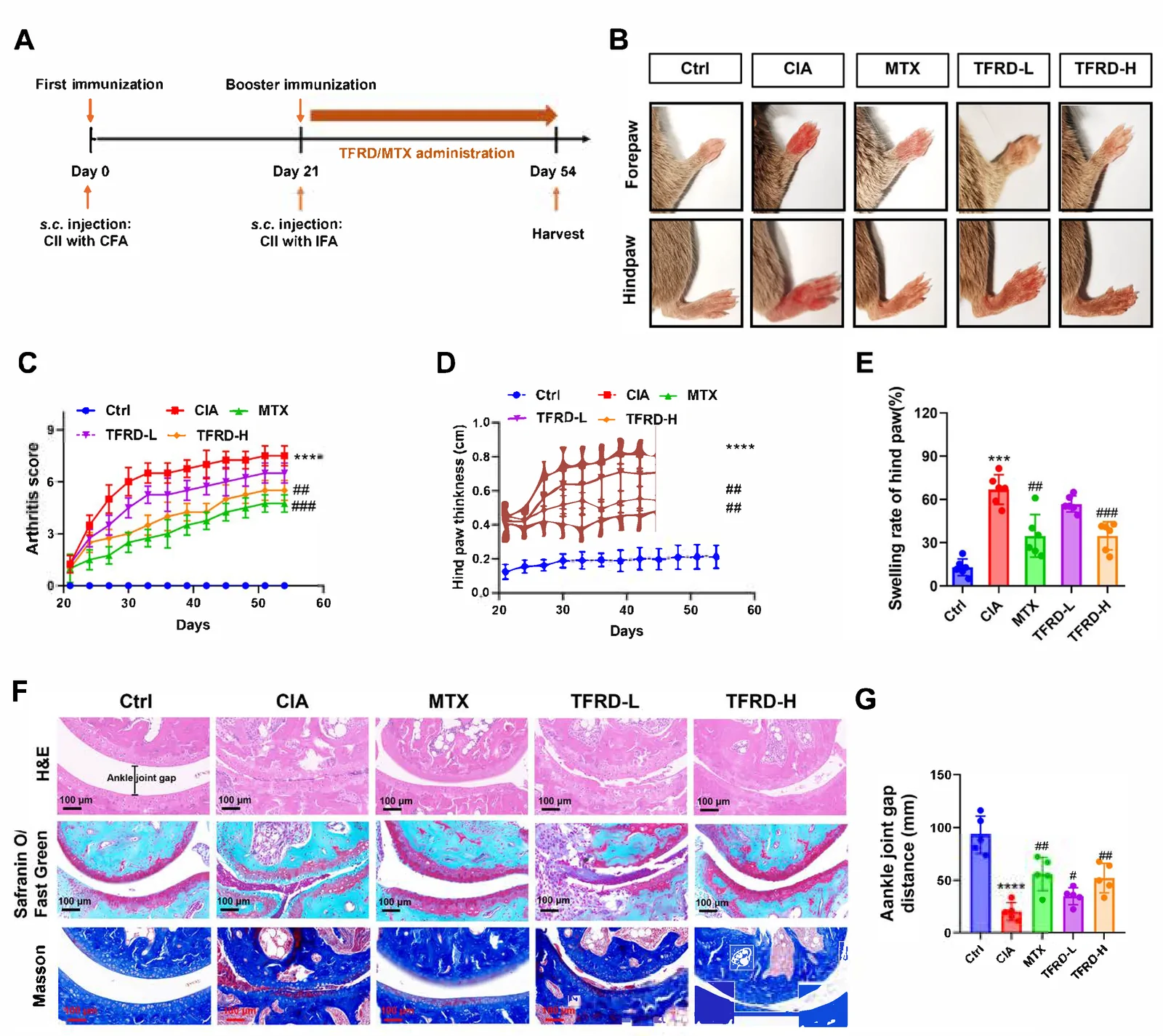
Significant efficacy of TFRD against joint damage in CIA mice. (**A**) Schematic diagram of immunization and treatment of CIA mice. (**B**) Representative images of paws and joints in mice on day 54. (**C**) Changes in arthritis scores of mice in each group. (**D**) Changes in paw thickness in each group. (**E**) Quantification of swelling inhibition rate in each group on day 54. (**F**) Representative hematoxylin–eosin, Safranin O/Fast green and Masson images of joint. (**G**) Quantitative analysis of ankle joint gap distance of mice in each group. Data are presented as means ± SDs; *N* = 6 mice for each group. Symbols *** and **** indicate *p* < 0.001 and *p* < 0.0001 compared with Ctrl group; symbols #, ## and ### indicate *p* < 0.05, *p* < 0.01 and *p* < 0.001 compared with CIA group.

To investigate the effect of TFRD on arthritis, we conducted a series of in vivo experiments using CIA mice. After booster immunization, the mice were administrated different concentrations of TFRD for 33 days. Arthritis severity and paw swelling were assessed at three-day intervals throughout the treatment period (Figure 1A). The hind limbs of mice in the CIA group displayed marked erythema and edema, primarily localized to the ankle joints and digits. In contrast, both the MTX and TFRD treatment groups exhibited varying degrees of attenuation in joint swelling and digital erythema (Figure 1B). The arthritis scores in the TFRD-H and MTX groups were significantly reduced compared to those in the CIA group during treatment (Figure 1C). In addition, the hind-paw thicknesses in the TFRD-H and MTX groups were also decreased compared to those in the CIA group (Figure 1D). Quantitatively, the swelling rates of mice in the MTX and TFRD-H groups were decreased by 47.9 ± 5% and 40.1 ± 8% on day 54, respectively (Figure 1E). Next, we evaluated the pathomorphological changes in the ankle joint by utilizing H&E, Safranin O/Fast Green and Masson’s trichrome staining. Compared to the Ctrl group, synovial hyperplasia, massive inflammatory cell infiltration and evident articular cartilage erosion were observed in the CIA group. However, MTX and TFRD-H treatment remarkably alleviated these pathological injuries (Figure 1F). Furthermore, the ankle joint distance was decreased in CIA mice compared to that in Ctrl mice, which could be reversed by MTX and TFRD treatment (Figure 1G). Together, these results indicated that TFRD have good therapeutic effects against RA in CIA mice.

### 2.2. TFRD Suppress Bone Destruction and Osteoclastogenesis in CIA Mice

**Figure 2:**
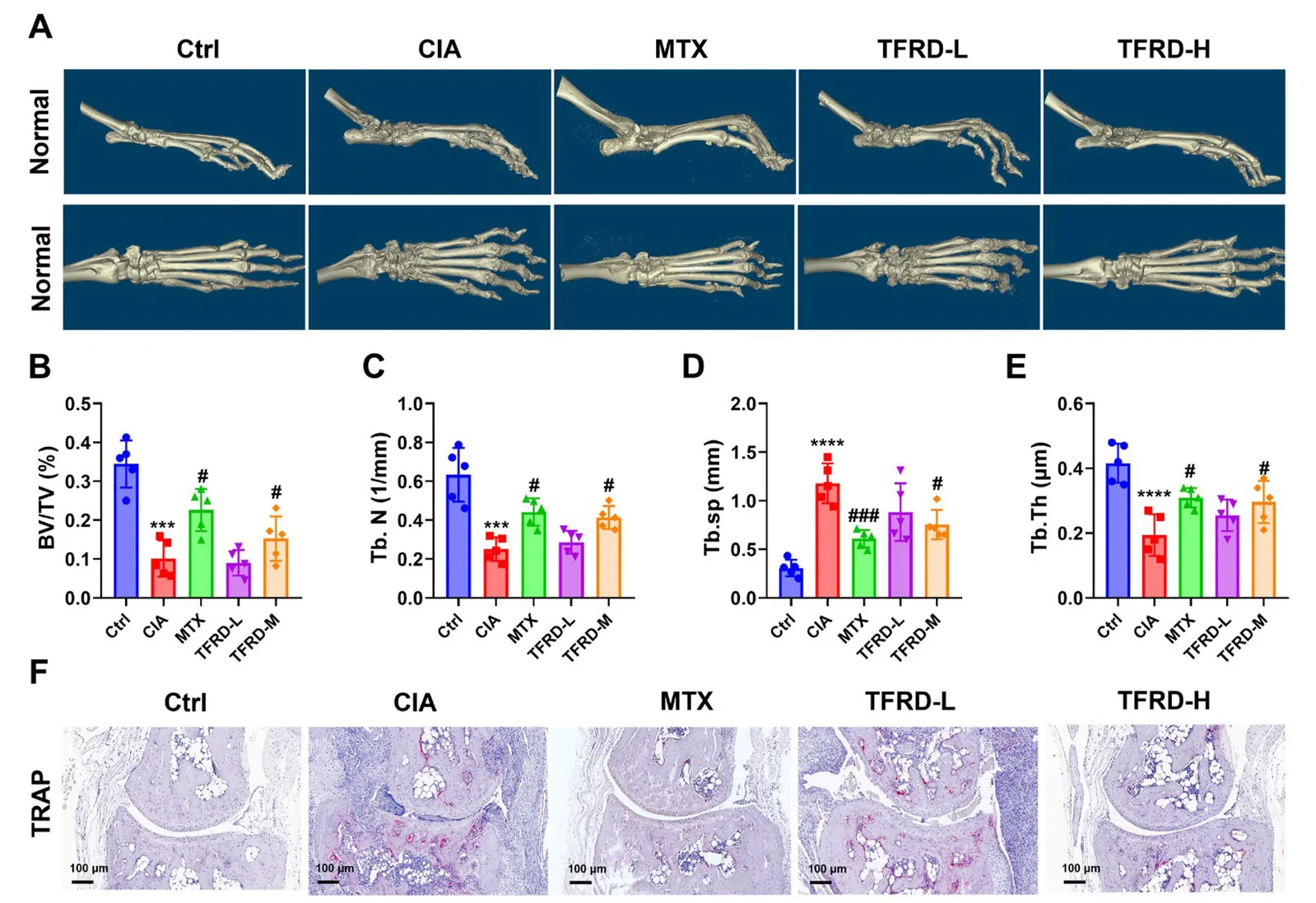
Effect of TFRD on bone destruction and osteoclastogenesis in CIA mice. (**A**) Representative micro-CT images of ankle joints. (**B**–**E**) Quantitative analysis of BV/TV (**B**), Tb.N (**C**), Tb.Sp (**D**) and Tb.Th (**E**). (**F**) Representative images of TRAP staining of joints. Data are presented as means ± SDs; *N* = 6 mice for each group. Symbols *** and **** indicate *p* < 0.001 and *p* < 0.0001 compared with Ctrl group; symbols # and ### indicate *p* < 0.05 and *p* < 0.001 compared with CIA group.

Next, we evaluated the osteoprotective effects of TFRD in CIA mice. The micro-CT images revealed markedly aggravated articular surface irregularity and bone erosion in CIA mice compared with the Ctrl group, and these damages were reduced in the TFRD and MTX groups (Figure 2A). In addition, the mice in the CIA group exhibited significant reductions in their bone volume fraction (BV/TV), trabecular number (Tb.N), and trabecular thickness (Tb.Th), alongside an increase in trabecular separation (Tb.Sp) relative to the Ctrl group. However, TFRD-H and MTX treatment markedly rescued the BV/TV, Tb.N, and Tb.Th and reduced the value of the Tb.Sp relative to the CIA group (Figure 2B–E). Furthermore, we investigated the contents of osteoclasts in bone tissue by TRAP staining. The results showed that TRAP+ cells were markedly increased in the CIA group compared to in the Ctrl group. TFRD-H and MTX treatment both led to reduced TRAP+ cells relative to the CIA group (Figure 2F). These results suggested that TFRD alleviated bone destruction and the inhibition of osteoclastogenesis.

### 2.3. TFRD Attenuated Population of Pro-Inflammatory Macrophages in CIA Mice

To investigate whether TFRD could directly regulate osteoclast differentiation, we isolated bone marrow-derived macrophages (BMDMs) and applied RANKL to induce an osteoclast differentiation experiment in vitro. The results demonstrated that the cell viability of BMDMs showed no significant difference compared with the control group after treatment with 0–600 μg/mL TFRD for 24 h (Figure S3A). Furthermore, the proportion of TRAP+ cells showed no significant difference between TFRD-treated groups at various concentrations and the vehicle group (Figure S3B,C). Meanwhile, quantitative PCR analysis indicated that TFRD treatment did not affect the expression of osteoclast differentiation-related genes in BMDMs (Figure S3D). These findings suggested that TFRD does not directly modulate osteoclast differentiation.

**Figure 3:**
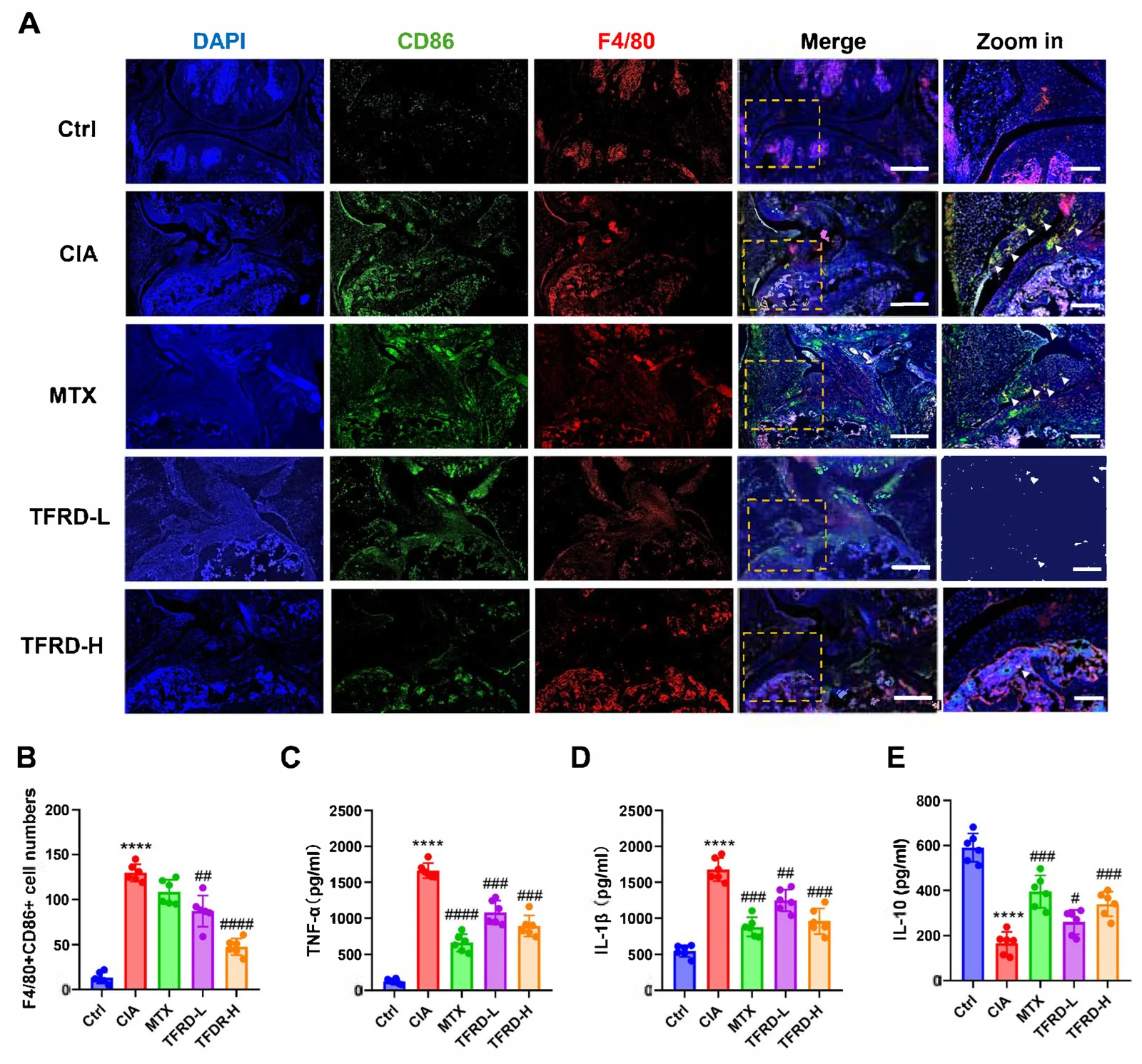
Effect of TFRD on population of pro-inflammatory macrophages in CIA mice. (**A**) Representative immunofluorescence images of CD86 (green), F4/80 (red) and DAPI (blue) in ankle joints from each group; yellow box represents volume of interest (VOI); scale bar = 200 μm. (**B**) Quantitative analysis of F4/80 + CD86+ macrophages in VOI from subfigure (**A**). (**C**–**E**) Levels of TNF-α (**C**), IL-1β (**D**) and IL-10 (**E**) in sera from each group. Data are presented as means ± SDs; *N* = 6 mice for each group; **** *p* < 0.0001 compared with Ctrl group; symbols #, ##, ###, and #### indicate *p* < 0.05, *p* < 0.01, *p* < 0.001, and *p* < 0.0001 compared with CIA group.

The population of pro-inflammatory macrophages is elevated in the synovial tissue during RA [7]. Numerous studies have demonstrated that pro-inflammatory macrophage-derived cytokines contribute to synovial inflammation and promote osteoclastogenesis [26,27]. Therefore, we elucidated the effect of TFRD on macrophage polarization in CIA mice. Compared to the Ctrl group, the number of pro-inflammatory macrophages (F4/80 + CD86 +) was markedly increased in the CIA group but was significantly decreased by TFRD-L and TFRD-H treatment (Figure 3A,B). Furthermore, the protein levels of inflammatory cytokines, such as TNF-α and IL-1β, were lower in the sera of animals from the TFRD-L and TFRD-H treatment groups compared to those in the CIA group (Figure 3C,D). In addition, TFRD application increased the level of IL-10, a well-known anti-inflammatory cytokine, relative to CIA mice (Figure 3E). These data indicated that TFRD inhibited the pro-inflammatory polarization of macrophages in joints during RA.

### 2.4. TFRD Inhibited the Pro-Inflammatory Polarization of Cultured Macrophages in Vitro

**Figure 4:**
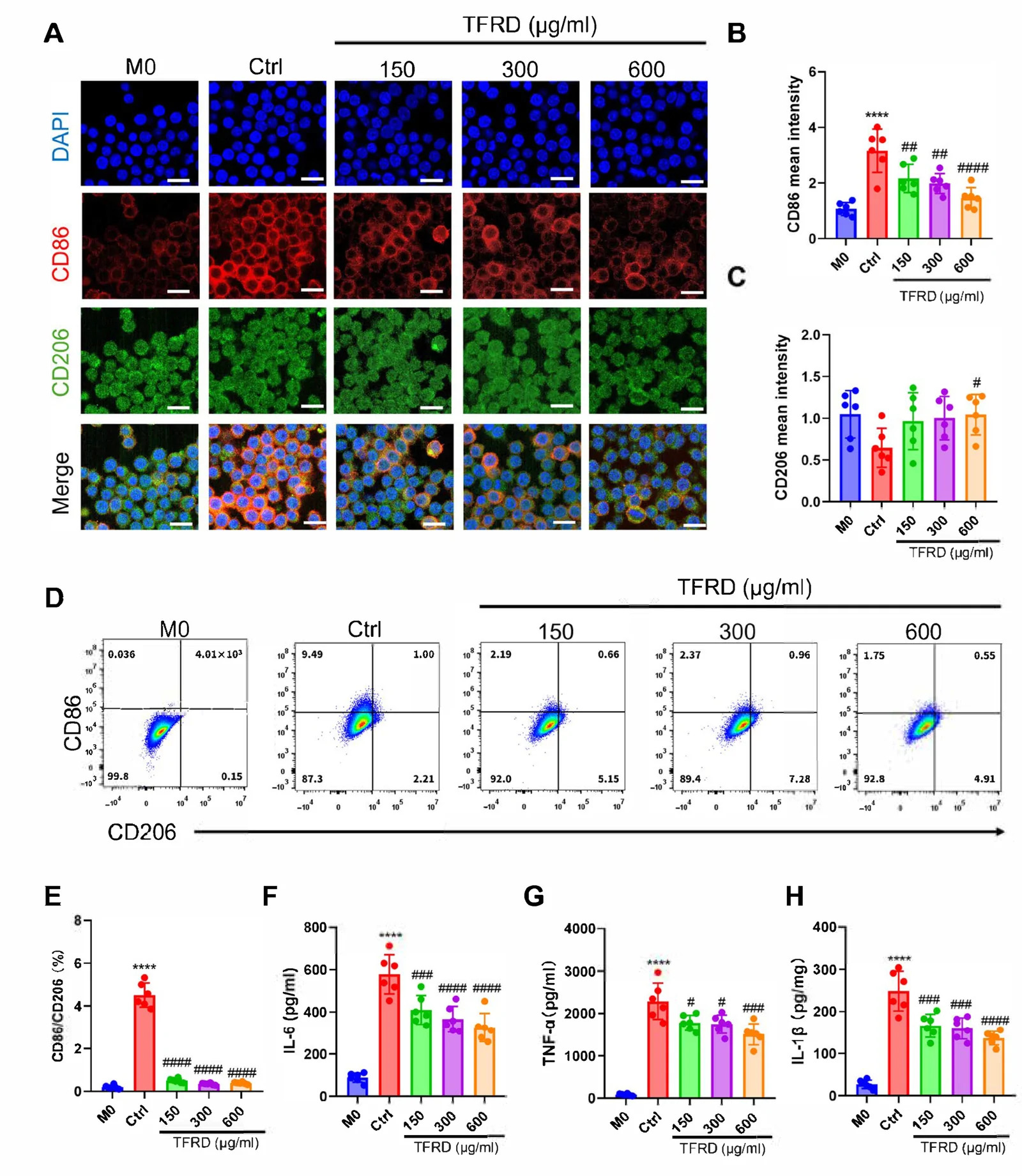
Effect of TFRD on polarization of RAW264.7 cells in vitro. (**A**) Representative immunofluorescence images of CD86 (red), CD206 (green) and DAPI (blue) in RAW264.7 cells; scale bar = 20 μm. (**B**,**C**) Quantitative analysis of CD86 and CD206 intensity from A. (**D**,**E**) Flow cytometry analysis of CD86/CD206 proportion in RAW264.7 cells. (**F**–**H**) Levels of IL-6 (**F**), TNF-α (**G)** and IL-1β (**H**) in culture supernatant from RAW264.7 cells. In all experiments, RAW264.7 cells were treated with LPS (100 ng/mL) and TFRD for 24 h. Data are presented as means ± SDs; *N* = 6 independent biological experiments for each group; **** *p* < 0.0001 compared with M0 group; symbols #, ##, ###, and #### indicate *p* < 0.05, *p* < 0.01, *p* < 0.001, and *p* < 0.0001 compared with Ctrl group.

Next, we examined the potential influence of TFRD on macrophage polarization by conducting a series of experiments on RAW264.7 cells with TFRD intervention in vitro. The immunofluorescence results showed that the mean intensity of CD86 was markedly increased in RAW264.7 cells following 24 h of LPS (100 ng/mL) stimulation, whereas this elevation was suppressed by TFRD treatment (Figure 4A,B). In addition, the mean intensity of CD206 exhibited a declining trend upon LPS stimulation without statistical significance (*p* = 0.0687), whereas treatment with 600 μg/mL TFRD reversed this reduction (Figure 4C). Flow cytometry results showed that the CD86/CD206 proportions were significantly increased after LPS stimulation and were markedly decreased by TFRD treatment (Figure 4D,E). Further, the result from the ELISA showed that LPS stimulation significantly upregulated the concentrations of pro-inflammatory cytokines (IL-6, TNF-α and IL-1β) in the cell supernatant, which were all reduced in the TFRD administration groups (Figure 4F–H). These results revealed that TFRD attenuated the pro-inflammatory macrophage phenotype and downregulated the expression of pro-inflammatory cytokines.

### 2.5. TFRD Altered the Expression of HIF-1α in CIA Mice

The network pharmacology analysis was performed to explore the potential mechanisms by which TFRD exerts therapeutic effects against RA. Through database analysis, 147 overlapping target genes were obtained between TFRD and RA (Figure S4A). The PPI network showed 89 high-confidence targets (Figure S4B). Additionally, based on the degree values of the network analysis, HIF-1α was screened out as a potential core target for subsequent experimental validation (Figure S4C). The GO enrichment was analyzed using the DAVID database (Figure S4D). Further, KEGG pathway enrichment showed that the HIF-1α signaling pathway could be regulated by TFRD administration (Figure S4E).

**Figure 5:**
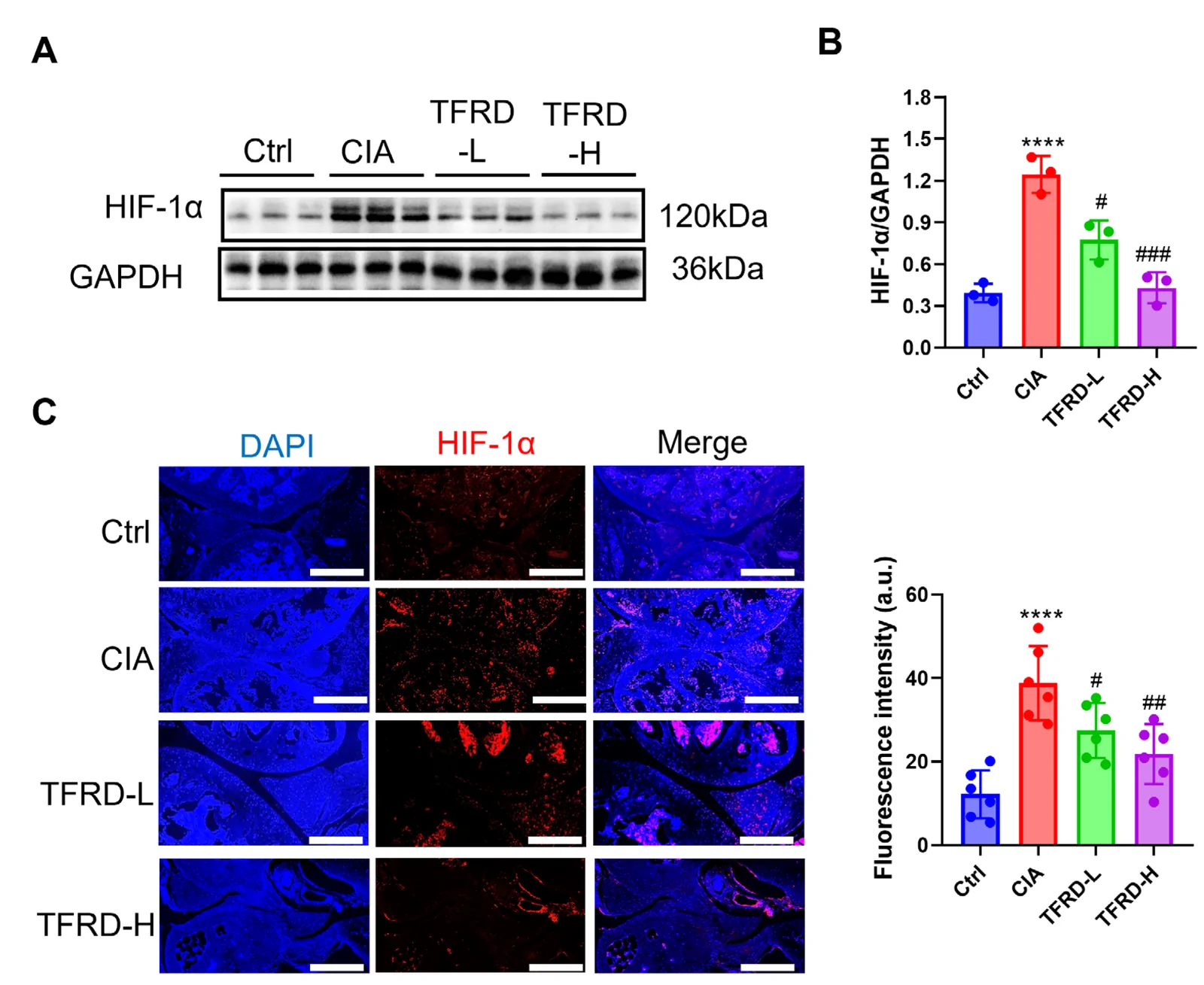
Effect of TFRD on HIF-1α expression levels in CIA mice. (**A**) Western blot analysis of HIF-1α protein expression in ankle joints of CIA mice. (**B**) Quantitative analysis of relative expression of HIF-1α; *N* = 3 mice for each group. (**C**) Immunofluorescence images and quantitative analysis of HIF-1α in ankle joints; scale bar = 200 μm. Data are presented as means ± SDs; *N* = 6 mice for each group. **** *p* < 0.0001 compared with Ctrl group; symbols #, ##, and ###, indicate *p* < 0.05, *p* < 0.01, and *p* < 0.001 compared with CIA group.

The elevated expression of HIF-1α has been confirmed in RA patients [28,29], and recent evidence has verified that this alteration is correlated with an increased abundance of pro-inflammatory macrophages [21]. Thus, we measured the expression of HIF-1α in CIA mice to investigate the effect of TFRD more precisely. Results of the Western blotting showed that the protein expression of HIF-1α in the joints of CIA mice was significantly upregulated compared to that in the joints of Ctrl mice, and TFRD treatment reduced it in a dose-dependent manner (Figure 5A,B). Immunofluorescence results also showed that the intensity of HIF-1α was markedly decreased in the TFRD administration group compared to that in the CIA group (Figure 5C). These results demonstrated that TFRD treatment suppressed the HIF-1α expression in the joints of CIA mice.

### 2.6. TFRD Inhibited HIF-1α Expression and Downregulated Glycolysis in Macrophages in Vitro

**Figure 6:**
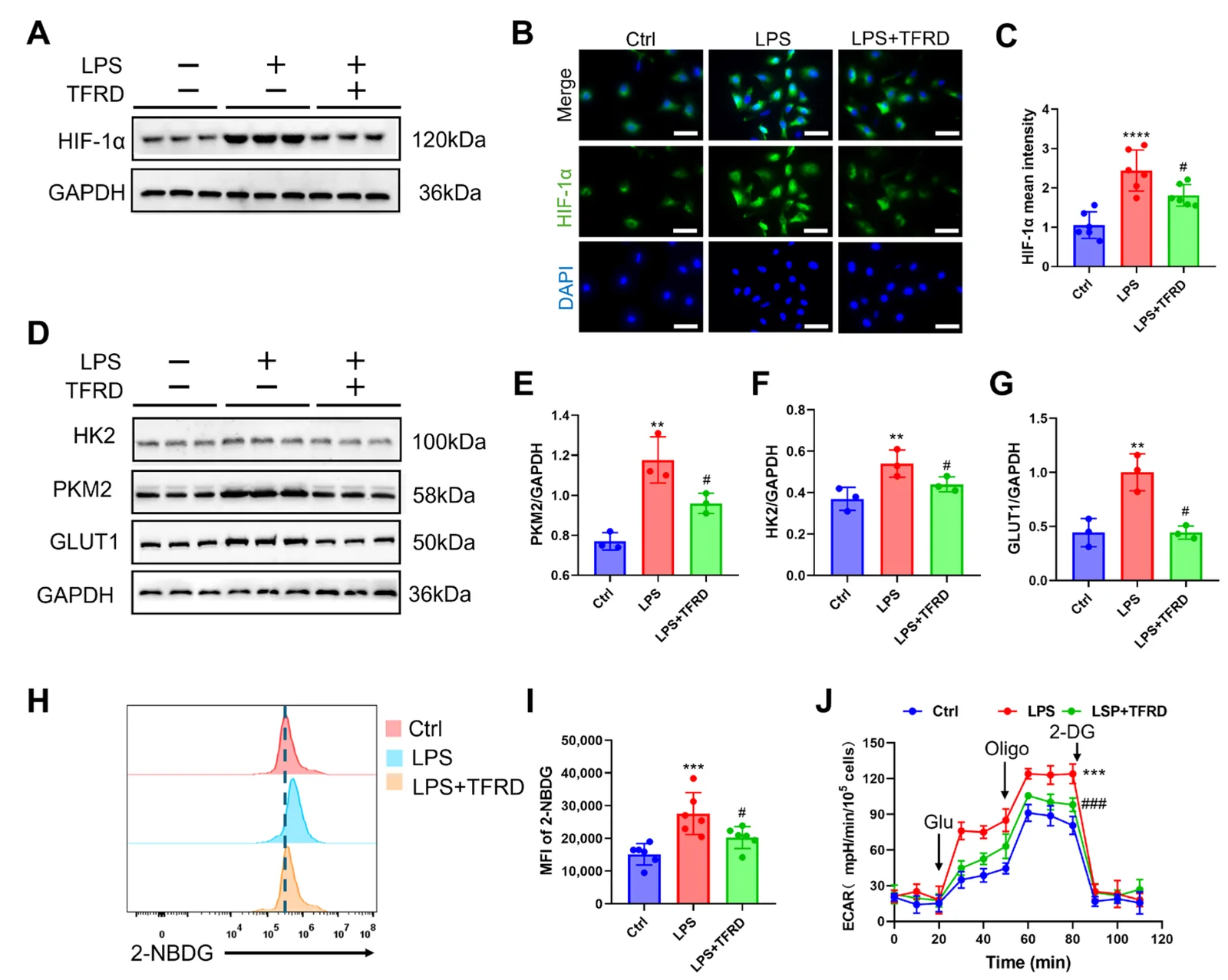
Effect of TFRD on HIF-1α expression and glycolysis in RAW264.7 cells. (**A**) Western blot analysis of protein expression level of HIF-1α in RAW264.7 cells. (**B**) Representative immunofluorescence images of HIF-1α (green) and DAPI (blue) in RAW264.7 cells; scale bar = 20 μm. (**C**) Quantitative analysis of intensity of HIF-1α from B. (**D**–**G**) Representative image of Western blot and quantitative analysis of HK2, PKM2 and GLUT1. (**H**–**I**) Flow cytometry images and quantitative analysis of 2-NBDG in RAW264.7 cells. (**J**) ECAR analysis. In all experiments, RAW264.7 cells were treated with LPS (100 ng/mL) and TFRD (300 μg/mL) for 24 h. MFI: mean fluorescence intensity; dash line: threshold of MFI. Data are presented as means ± SDs; *N* = 3–6 independent biological experiments for each group. Symbols **, ***, and **** indicate *p* < 0.01, *p* < 0.001, and *p* < 0.0001 compared with Ctrl group. Symbols # and ### indicate *p* < 0.05 and *p* < 0.001 compared with LPS group.

Metabolic reprogramming is indispensable for macrophage pro-inflammatory polarization [9], and HIF-1α has been demonstrated to facilitate pro-inflammatory polarization by boosting glycolysis in macrophages [13,14,21]. Therefore, we examined the potential influence of TFRD on HIF-1α expression and glycolysis in macrophages. The results from the Western blot and immunofluorescence imaging showed that the protein expression of HIF-1α was markedly upregulated after LPS induction and was reduced by TFRD administration (Figure 6A–C). In addition, LPS stimulation upregulated the protein expression level of rate-limiting enzymes participating in glycolysis, including HK2, PKM2 and GLUT1. However, TFRD administration reversed this elevation (Figure 6D–G). We detected the glucose uptake through 2-NBDG, a glucose analog, in cultured macrophages. Compared to the Ctrl group, the mean fluorescence intensity of 2-NBDG was increased in the LPS stimulation group and downregulated by TFRD application (Figure 6H,I). Further, we examined the extracellular acidification rate (ECAR) in polarized macrophages. The results showed that LPS stimulation elevated glycolysis in RAW264.7 cells, while TFRD application abrogated this LPS-mediated glycolysis upregulation (Figure 6J). Overall, these results indicate that the downregulation of HIF-1α expression and glycolysis was involved in the TFRD-mediated suppression of macrophage pro-inflammatory polarization.

### 2.7. TFRD Downregulated Glycolysis and Macrophage Pro-Inflammatory Polarization Partly Through Suppressing HIF-1α

**Figure 7:**
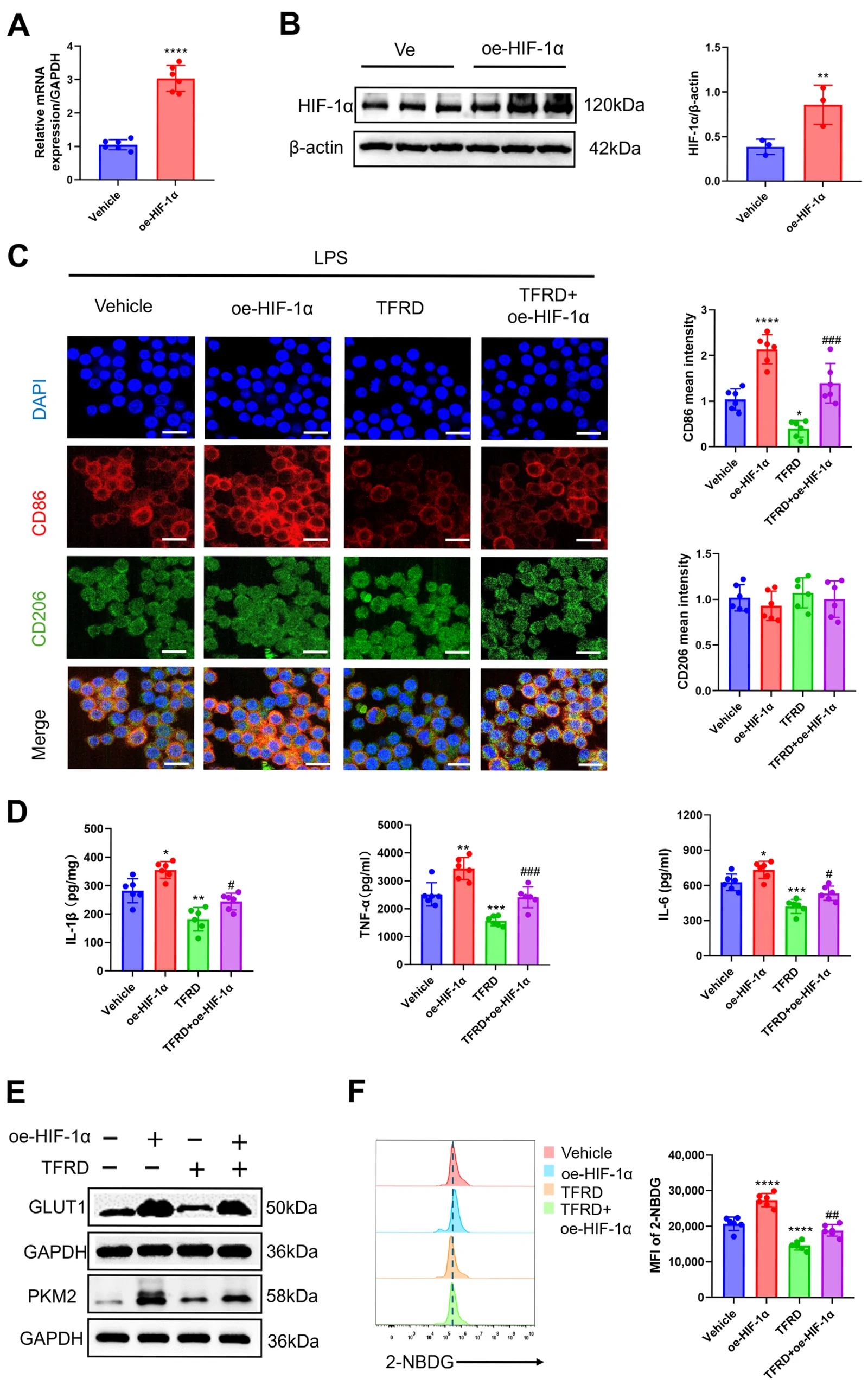
TFRD inhibited macrophage pro-inflammatory polarization by downregulating glycolysis levels via suppression of HIF-1α. (**A**) RT-PCR analysis of mRNA expression of HIF-1α after 48 h of plasmid transfection in RAW264.7 cells. (**B**) Western blot analysis of protein expression of HIF-1α. (**C**) Immunofluorescence analysis of CD86 (red), CD206 (green) and DAPI (blue); scale bar = 20 μm. (**D**) ELISA analysis of levels of IL-6, TNF-α and IL-1β in culture supernatant. (**E**) Western blot analysis of protein expression of GLUT1 and PKM2. (**F**) Flow cytometry images and quantitative analysis of 2-NBDG. In all experiments, RAW264.7 cells were treated with vehicle or HIF-1α overexpression plasmid for 48 h and were then treated with LPS (100 ng/mL) and TFRD (300 μg/mL) for 24 h. MFI: mean fluorescence intensity; dash line: threshold of MFI. Data are presented as means ± SDs; *N* = 3–6 independent biological experiments for each group. Symbols *, **, ***, and **** indicate *p* < 0.05, *p* < 0.01, *p* < 0.001, and *p* < 0.0001 compared with vehicle group. Symbols #, ##, and ### indicate *p* < 0.05, *p* < 0.01, and *p* < 0.001 compared with TFRD group.

To verify whether TFRD suppressed macrophage pro-inflammatory polarization and glycolysis by modulating HIF-1α, we overexpressed HIF-1α in RAW264.7 cells through plasmid transfection. Compared with the vehicle, the mRNA and protein expression levels of HIF-1α were all increased by 2-fold in Hif-1α overexpression macrophages (Figure 7A,B). Immunofluorescence analysis results showed that TFRD treatment efficiently downregulated LPS-induced pro-inflammatory marker (CD86) accumulation, but overexpression of HIF-1α significantly abrogated the inhibitory effects of TFRD on macrophage pro-inflammatory polarization in RAW264.7 cells (Figure 7C). In addition, the concentrations of pro-inflammatory cytokines, including IL-6, TNF-α and IL-1β, were all decreased in TFRD treatment macrophages under the LPS stimulation condition. However, HIF-1α overexpression partly blocked the inhibitory effect of TFRD on these cytokines (Figure 7D). Next, we studied whether HIF-1α participates in the suppression effect of TFRD on glucose metabolism in macrophages. The Western blot results showed that the protein expressions of GLUT1 and PKM2 were decreased in the TFRD treatment group and were blocked by HIF-1α overexpression (Figure 7E). Additionally, under LPS stimulation, TFRD treatment decreased the 2-NBDG intensity in RAW264.7 cells, while HIF-1α overexpression suppressed this inhibitory effect (Figure 7F). Thus, we conclude that TFRD regulates macrophage polarization partially by suppressing HIF-1α-mediated glycolysis.

### 2.8. TFRD Regulated the Macrophage Polarization-Associated Microbiota in CIA Mice

**Figure 8:**
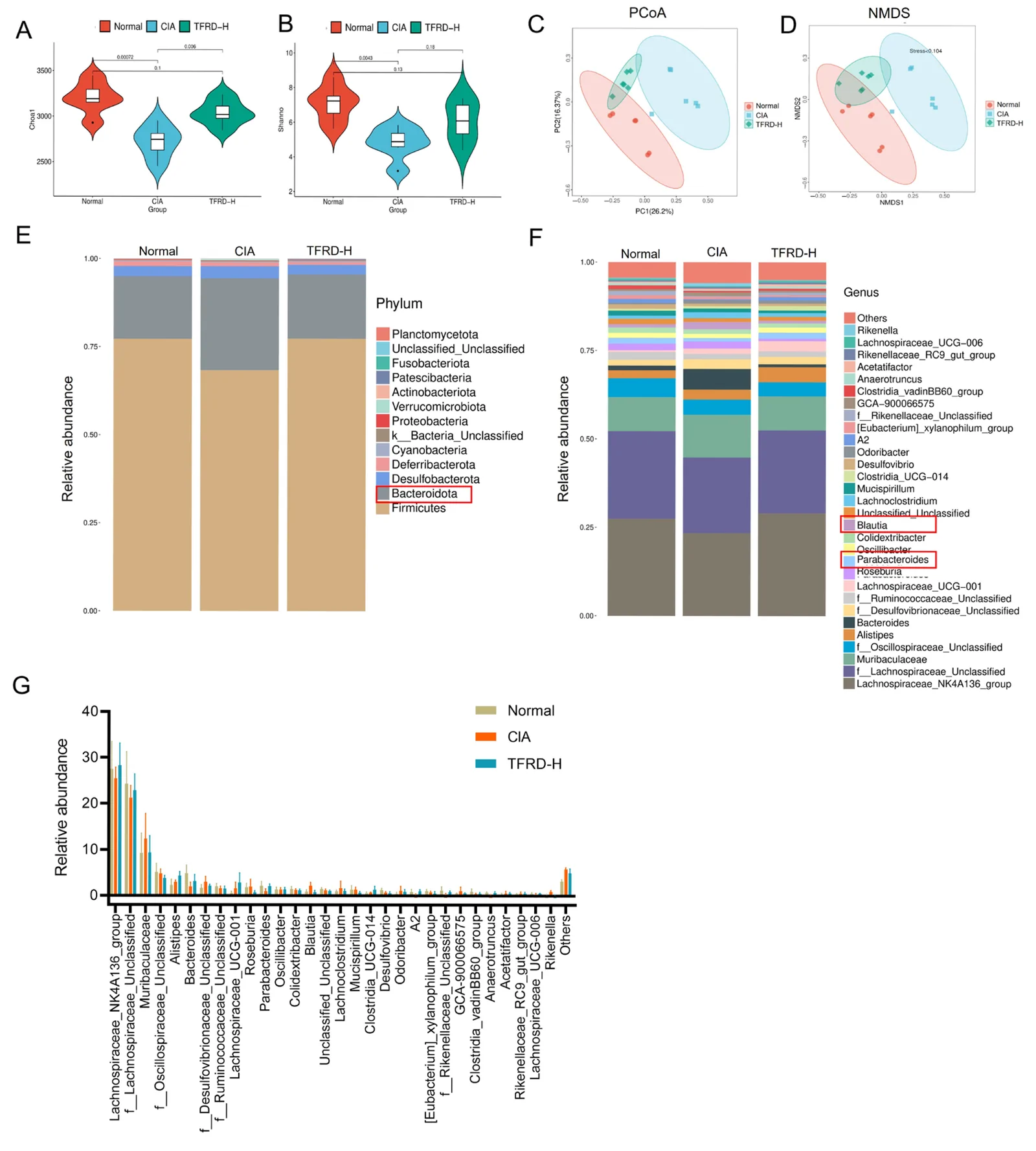
TFRD alters gut microbiota composition in CIA mice. (**A**,**B**) Alpha diversity of gut microbiota across groups. (**C**,**D**) Beta diversity analysis using PCA and PCoA. (**E**,**F**) Composition of gut microbiota at phylum and genus levels. (**G**) Comparison of relative microbial abundance at genus level. Data are presented as means ± SDs (*N* = 6 mice per group).

Altered gut microbiotas have been proven to indirectly participate in macrophage polarization through secreted metabolic products during RA [30,31,32]. Therefore, we performed 16S rRNA sequencing to analyze the microbial composition of the intestinal contents in CIA mice. Compared with the Ctrl group, the abundance and diversity of colonies were decreased in CIA mice, whereas treatment with high-dose TFRD significantly ameliorated this reduction (Figure 8A,B). PCoA and NMDs revealed that the samples from the control and CIA groups were clustered in two different areas, and the TFRD treatment group was clustered between the control and CIA groups, indicating that TFRD application improved the structure of colonies (Figure 8C,D). The differential analysis of the microbiota at the phylum level revealed that the abundance of *Bacteroides* was reduced in CIA mice but was recovered by TFRD treatment (Figure 8E). The differential analysis of the microbiota at the genus level revealed that TFRD increased the relative abundance of *Parabacteroides* and *Lachnospiraceae_NK4A136*, whereas they decreased the abundance of *Blautia* (Figure 8F,G). According to previous reports, Parabacteroides could modulate macrophage polarization by producing bile acids, and it has an anti-inflammatory effect during RA [30]. In addition, the elevated abundance of *Blautia* is associated with the excessive activation of Tfh cells in RA [33]. These results indicated that the altered abundance of gut microbiotas may be associated with the anti-inflammatory effect of TFRD in CIA mice.

## 3. Discussion

RA is a common autoimmune disease characterized primarily by persistent inflammation and bone destruction, with a prevalence of approximately 0.5–1.0% globally [34]. Although the exact pathogenesis of RA remains unclear, the abundant infiltration of pro-inflammatory macrophages in the joint synovium is considered to be a trigger of the persistent inflammation in RA [5,7]. The multiple pro-inflammatory cytokines continuously secreted by macrophages not only directly damage articular cartilage but also accelerate joint erosion by promoting osteoclastogenesis [27,35]. Natural products have previously demonstrated anti-inflammatory effects in experimental models, supporting the potential of phytochemical-rich preparations as sources of anti-inflammatory agents. Ethanolic extracts of different varieties of *Capsicum annuum* attenuated carrageenan-induced paw edema in rats and exhibited analgesic activity [36]. Likewise, methanolic extracts of *Cicer arietinum* demonstrated significant anti-inflammatory activity in the carrageenan-induced paw edema model, along with analgesic activity [37]. These findings provide a broader preclinical context for the anti-inflammatory activity of plant-derived preparations. TFRD, the major bioactive compounds extracted from the traditional Chinese Medicinal herb *Drynariae rhizoma*, have been widely utilized for treating bone disorder [38,39]. Recent studies have reported the therapeutic benefits of TFRD in RA [24,25], but it is unknown whether TFRD exert regulatory effects on macrophage polarization. In this study, we evaluated the beneficial effects of TFRD in the treatment of RA and demonstrated that TFRD effectively inhibit the pro-inflammatory polarization of macrophages. Mechanically, we found that the suppression of HIF-1α-associated glycolysis was partly involved in the inhibition of pro-inflammatory macrophage polarization induced by TFRD. Our findings offer novel insights into the anti-inflammatory effects of TFRD in the treatment of RA.

Osteoclasts are the principal cells involved in bone absorption and degradation in vivo. In patients with RA, excessive osteoclast activation contributes to bone destruction and leads to the irreversible loss of joint function [26,35]. In the present study, we found that TFRD treatment remarkably ameliorated bone erosion and reduced the number of TRAP-positive osteoclasts in CIA mice, suggesting the potent anti-osteoclastic activity of TFRD in vivo. However, further in vitro experiments revealed that TFRD treatment exerted no obvious influence on the RANKL-induced osteoclast differentiation in BMDMs. These findings indicate that TFRD suppressed pathological bone resorption in RA rather than directly interfered with osteoclast differentiation.

Macrophage polarization plays a critical role in modulating osteoclast activity [27]. Numerous studies have confirmed that pro-inflammatory macrophages facilitate the maturation of osteoclasts through the production of TNF-α, IL-6 and IL-1β, whereas anti-inflammatory macrophages inhibit this process by secreting IL-4 and IL-10 [40]. During RA progression, the homeostatic balance between pro-inflammatory and anti-inflammatory macrophages is disrupted [41]. The abundant accumulation of pro-inflammatory macrophages in synovial fluid and joint tissues has been shown to be involved in excessive osteoclast activity, and downregulating pro-inflammatory macrophages could suppress this process in RA [26,42]. Here, we found that TFRD markedly decreased the abundance of pro-inflammatory macrophages but failed to directly regulate osteoclast differentiation. Combined with the fact that pro-inflammatory macrophages upregulate osteoclastogenesis in RA, we hypothesis that the anti-osteoclastogenic effect of TFRD occurs partly through modulation of pro-inflammatory macrophages. Although macrophages represent the most abundant immune cells in the RA synovium [43,44], T lymphocytes, B lymphocytes, synovial fibroblasts and neutrophils have all been reported to participate in the inflammatory process of RA by secreting inflammatory mediators and cytokines [45]. Recent studies have demonstrated that TFRD could inhibit Th17 cell differentiation by modulating the intestinal microbiota in RA. Combined with our findings, this suggests that the anti-arthritic efficacy of TFRD exhibits multi-target characteristics.

Metabolic reprogramming toward aerobic glycolysis is a hallmark of macrophage pro-inflammatory polarization [9,13,46]. HIF-1α, a critical transcription factor involved in cellular adaptation to hypoxia, could regulate the expression of glycolytic rate-limiting enzymes, including PKM2, HK2 and GLUT1, leading to enhanced glycolysis and promoting macrophage pro-inflammatory polarization [13,14,16]. Elevated HIF-1α expression has been widely detected in the synovial tissues and sera of RA patients, and this molecular alteration is closely linked to multiple pathological processes of RA [21,47]. It has been reported that sera from RA patients amplifies the glycolysis–HIF-1α loop, which promotes macrophage pro-inflammatory polarization and IL-1β production [21]. In addition, inhibition of the ERK/HIF-1α/GLUT1 axis induces metabolic reprogramming in macrophages, facilitating the shift from a pro-inflammatory to anti-inflammatory phenotype in RA [12]. These results indicate that HIF-1α could serve as a therapeutic target to alleviate pro-inflammatory macrophages in RA. In our study, we demonstrated that TFRD inhibited HIF-1α expression in the joints of CIA mice. Furthermore, TFRD suppressed glycolysis and subsequent pro-inflammatory polarization partly by downregulating HIF-1α expression in cultured macrophages. These results indicate that TFRD reduced the accumulation of pro-inflammatory macrophages in RA joints, and that the suppression of HIF-1α-induced glycolysis is partially involved in this process. Naringin, a major component of TFRD, could reduce glycolysis in colon cancer cells by suppressing the transcriptional activity of HIF-1α [48]. Thus, naringin is a candidate constituent that may contribute to the observed effects and warrants direct investigation in future studies. In addition, the robust production of reactive oxygen species (ROS) and mitochondrial oxidative stress induced by HIF-1α also participates in pro-inflammatory macrophage polarization [49]. Recent studies have reported that TFRD could reduce ROS and mitochondrial oxidative stress in RA [50]. Thus, HIF-1α-mediated oxidative stress may also be regulated by TFRD and contribute to the modulation of macrophage polarization in RA. Beyond driving macrophage pro-inflammatory polarization, overexpressed HIF-1α also contributes to synovial angiogenesis, inflammatory cell infiltration and cartilage damage in RA [19,20,28]. Thus, the downregulation of HIF-1α induced by TFRD application may also exert direct protective effects against synovial angiogenesis and cartilage destruction.

The gut microbiota has emerged as a critical regulator of joint inflammation via the gut–joint axis by secreting hormones, short-chain fatty acids and bile acids in RA [51,52]. Increased abundance of *Prevotella copri* has been proven to be related to elevated Th17 cells in RA patients [53]. Mechanically, *Prevotella copri* could promote Th17 cell differentiation and exacerbate joint inflammation in RA mice [24]. Additionally, Parabacteroides, a typical anti-inflammatory genus, could promote macrophage anti-inflammatory polarization in joints by producing iso lithocholic acid. The reduced abundance of Parabacteroides in RA contributes to the disrupted pro-inflammatory/anti-inflammatory balance [30]. In this study, 16S rRNA sequencing analysis revealed the gut microbiota dysbiosis in CIA mice, which was partially ameliorated by TFRD application. We found that the relative abundances of *Parabacteroides* and *Lachnospiraceae_NK4A136* decreased in CIA mice, while harmful taxa such as *Blautia* expanded, which is consistent with previous reports [30,33,54]. As per previous reports, *Parabacteroides* is linked to macrophage anti-inflammatory polarization in joints via bile acid production during RA progression [30]. *Blautia* could promote Tfh activation, leading to exacerbated inflammation in RA [33]. In this study, we found that TFRD administration markedly upregulated the abundance of *Parabacteroides* and downregulated the abundance of *Blautia*, suggesting a correlation between gut microbiota shifts and the beneficial effect of TFRD in RA treatment. The causal involvement of the gut microbiota in the efficacy of TFRD requires additional mechanistic studies. In addition, the relatively small sample size (n = 6 per group) for gut microbiota analysis may limit statistical power. Independent experiments with larger sample sizes are therefore required in future studies to validate these speculations.

## 4. Materials and Methods

### 4.1. Animals

Male DBA/1J and C57BL/6 mice, weighing 20 ± 2 g and aged between 8 and 10 weeks, were obtained from SPF Biotechnology Co., Ltd. (Beijing, China; license number SCXK 2019-0010). The animals were maintained under standardized conditions, including a 12-h light/dark cycle with unrestricted access to food and water. All experimental protocols involving the animals were performed in strict compliance with the guidelines approved by the Committee on Animal Care and Use of the Institute of Traditional Chinese Medicine Health Industry, China Academy of Chinese Medical Sciences (Animal Ethics Committee approval number: 2025015).

### 4.2. Identification of Active Ingredients in TFRD

The TFRD employed in this study were provided in capsule form by Qihuang Pharmaceutical Manufacturing Co., Ltd. (Beijing, China) (QiangGu capsule, 0.25 g per capsule; National Approval No. Z20030007; Item No. 04,080,081), with each capsule containing 0.18 g of TFRD. This preparation is a state-approved standardized total flavonoid formulation derived from *Drynariae rhizoma* and has been extensively utilized in pharmacological research [24,50,55]. It is reported that the principal marker compounds naringin and neoeriocitrin were present at approximately 213.72 mg/g and 112.46 mg/g, respectively [56]. A single batch of TFRD was consistently used for both in vivo and in vitro experiments throughout this study. Prior to use, we opened the capsule shells, weighed and dissolved the TFRD in purified water, and filtrated them through a 0.22 μm membrane filter to obtain a working solution. The prepared TFRD solutions were analyzed via ultra-performance liquid chromatography coupled with mass spectrometry (UPLC-MS) (Waters Corporation, Milford, MA, USA), in accordance with previously established protocols [39].

Chromatographic separation was performed using a Waters ACQUITY UPLC HSS T3 column (2.1 mm × 100 mm, 1.8 μm) (Waters Corporation, Milford, MA, USA) and operated at a flow rate of 300 μL/min, with a column temperature of 45 °C. The mobile phase consisted of 0.1% formic acid in water (solvent A) and acetonitrile (solvent B). The gradient elution program was executed as follows: 5% B from 0 to 1.5 min; 5–15% B from 1.5 to 10 min; 15–95% B from 10 to 30 min; maintained at 95% B from 30 to 35 min; decreased from 95% to 5% B between 35 and 36 min; and held at 5% B from 36 to 40 min.

Mass spectral data were acquired in both positive- and negative-ion modes using a quadrupole time-of-flight mass spectrometer X500R QTOF system (AB SCIEX, Framingham, MA, USA) equipped with an electrospray ionization (ESI) source. The mass spectrometry parameters were set as follows: nebulizer gas at 55 psi; heater gas at 55 psi; curtain gas at 35 psi; collisionally activated dissociation gas (CAD) at 7 psi; turbo spray temperature at 550 °C; ion spray voltage at −4500 V; declustering potential (DP) at 60 V; and collision energy (CE) spread at 10 eV. The mass range was configured from 100 to 1500 Da for the TOF MS analysis and from 50 to 1500 Da for the TOF MS/MS analysis. A dynamic background subtraction strategy employing an information-dependent acquisition (IDA) was utilized. Ions previously excluded were set to be omitted for a duration of 6 s following two occurrences. Data acquisition, processing, and analysis were performed using SCIEX OS software (version 3.0, AB SCIEX, Framingham, MA, USA).

### 4.3. Evaluation of Collagen-Induced Arthritis (CIA) in Mice and Drug Treatment

The collagen-induced arthritis (CIA) model was established as previously described [57]. In brief, male DBA/1J mice aged 8 weeks were subjected to a primary immunization at the base of the tail with 100 μL of an emulsion comprising equal volumes of 150 μg bovine type II collagen (Chondrex, Redmond, WA, USA) and complete Freund’s adjuvant (Chondrex, Redmond, WA, USA). A booster immunization was administered on day 21 via the same injection route, consisting of 75 μg bovine type II collagen emulsified with incomplete Freund’s adjuvant. Arthritis severity was evaluated using a validated clinical scoring system, where a score of 0 indicated normal conditions; 1 corresponded to mild swelling or erythema; 2 indicated moderate swelling and erythema extending from the ankle to the mid-foot; 3 represented pronounced swelling extending from the ankle to the metatarsal joints; and 4 denoted extensive swelling and erythema involving the ankle, foot, and digits [58,59].

Following the second immunization, the CIA mice were randomly divided into 5 groups (n = 6 for each group) as follows: the control group (Ctrl), model group (CIA), methotrexate group (MTX, 1.5 mg/kg, every 3 days), low-TFRD-dose group (TFRD-L, 150 mg/kg/day) and high-TFRD-dose group (TFRD-H, 300 mg/kg/day). The doses of TFRD were determined based on human-equivalent dose conversion and published reports [24,50]. Blinding was achieved by assigning unique identification numbers to animal cages. TFRD groups received daily gavage, while Ctrl and CIA groups were given sterile water for 33 consecutive days. All animals were euthanized on day 54. All outcome assessments were performed under blinded conditions. Semiquantitative scoring and image quantification were performed independently by two investigators who were blinded to the sample information.

### 4.4. Histological Analysis

Three fresh ankle joint specimens were fixed in 4% paraformaldehyde at 4 °C for 24 h, followed by decalcification in 0.5 M EDTA at 4 °C for a duration of 21 days. Subsequently, the samples were dehydrated through a graded alcohol series, embedded in paraffin, and sectioned into 5 μm thick slices. The tissue sections were then processed using protocols including hematoxylin and eosin (H&E) (G1120, Solarbio, Beijing, China), Masson’s trichrome staining solution (C0189S, Beyotime, Shanghai, China), and the Safranin O and Fast Green Cartilage Staining Kit (C0621S, Beyotime, Shanghai, China), according to the indicated instructions.

### 4.5. Microcomputed Tomography (Micro-CT) Analysis

Fresh ankle joints were harvested and subjected to high-resolution micro-CT scanning using a SkyScan 1172 system (Bruker, Billerica, MA, USA) at an isotropic voxel size of 12 μm. Scans were acquired at 60 kV and 100 μA with a 0.5 mm aluminum filter over a full 360° rotation at 0.5° angular increments, with two-frame averaging applied per projection. Three-dimensional (3D) reconstruction of the ankle joints was performed using Mimics 17 software (Materialise NV, Leuven, Belgium). Quantitative assessment of the trabecular bone microarchitecture parameters, including the total bone volume (BV), total tissue volume (TV), bone volume fraction (BV/TV), trabecular number (Tb.N), trabecular thickness (Tb.Th), and trabecular separation (Tb.Sp), was conducted using CTAn software (version 1.18) (Bruker, Kontich, Belgium).

### 4.6. Isolation of BMDMs and Osteoclast Differentiation

The bone marrow-derived macrophages (BMDMs) were isolated and the osteoclast differentiation assay was performed according to previously described methods [60]. Briefly, total bone marrow cells were isolated by flushing the bilateral femora and tibiae of C57BL/6 mice. Then, the isolated cells were incubated at 37 °C in 5% CO_2_ for 36 h. In order to generate BMDMs, nonadherent cells were gathered and cultured with the α-MEM medium (G4554, Servicebio, Wuhan, China) containing 40 ng/mL M-CSF (GB310046, Servicebio, Wuhan, Hubei, China), 10% fetal bovine serum (G8003, Servicebio, Wuhan, China), and 1% penicillin–streptomycin (G4003, Servicebio, Wuhan, China) for 48 h. For osteoclast differentiation, the adherent BMDMs were treated with an α-MEM supplement containing 50 ng/mL receptor activator of nuclear factor kappa-B ligand (RANKL, 90630ES, Yeasen, Shanghai, China), with different concentrations of TFRD for 8 days.

### 4.7. RT-qPCR Analysis

Total RNA was extracted from cells utilizing TRIzol reagent (catalog number 15596018, Thermo Fisher, Waltham, MA, USA) in accordance with established experimental procedures. First-strand complementary DNA (cDNA) synthesis was performed with 1 μg of total RNA employing oligo(dT) primers (catalog number K1622, Thermo Fisher, Waltham, MA, USA). Quantitative polymerase chain reaction (qPCR) assays were conducted in 20 µL reaction volumes comprising SYBR Green Master Mix (catalog number 4309155, Thermo Fisher, Waltham, MA, USA), the cDNA template, and gene-specific primers, as detailed in Supplementary Table S1. The thermal cycling protocol consisted of an initial denaturation at 95 °C for 10 min, followed by 40 amplification cycles of 95 °C for 10 s and 60 °C for 30 s. Relative gene expression levels were normalized to GAPDH and quantified using the comparative ΔΔCt method.

### 4.8. Immunofluorescence Imaging

For tissue imaging, the fresh joints were fixed in 4% paraformaldehyde at 4 °C for 24 h, decalcified, embedded in OCT compound and sectioned. The slices were incubated with 20% normal goat serum for 1 h to block antigenic epitope. Then, the samples were stained with primary antibodies against HIF-1α, CD86 and F4/80 (Supplementary Table S2) at 4 °C for 12 h. After washing, the slices were incubated with Alexa powder 488- or 594-conjugated secondary antibodies. For cell imaging, the RAW264.7 cells were seeded on glass coverslips in 24-well plates, with a density of 1 × 10^5^ cells per well. After overnight incubation, cells were induced with LPS (100 ng/mL) and different concentrations of TFRD for 24 h. After the experiments, cells were fixed, permeabilized and blocked routinely, and they were then incubated with primary antibodies against CD86 and CD206 (Supplementary Table S2) at 4 °C for 12 h, followed by fluorescent secondary antibodies. All slices were mounted with DAPI-Fluorom-G (0100--20, SouthernBiotech, Birmingham, AL, USA) and coverslipped. The images were acquired by utilizing a Leica STELLARIS confocal microscope (Leica, Wetzlar, Germany) and analyzed using the manufacturer’s proprietary software.

### 4.9. Network Pharmacology

The bioactive components of total TFRD were extracted from the Traditional Chinese Medicine Systems Pharmacology Database and Analysis Platform (TCMSP; https://old.tcmsp-e.com (accessed on 19 December 2025)). Flavonoids retrieved from the TCMSP database were screened based on the criteria of an oral bioavailability (OB) ≥ 30% and a drug-likeness (DL) ≥ 0.18, according to the recommendation of the TCMSP platform and previous reports [61]. These screening criteria may exclude certain compounds with potential biological activity that exhibit poor predicted pharmacokinetic properties. Therefore, the identified compounds should be regarded as candidate active ingredients rather than a comprehensive representation of all pharmacologically relevant components. Potential molecular targets of TFRD constituents were compiled from the TCMSP, SwissTargetPrediction, and STITCH databases. Canonical SMILES strings of candidate compounds were obtained from PubChem. Targets with confidence scores above 0.4 were selected for further investigation. Genes associated with rheumatoid arthritis (RA) were retrieved from the GeneCards and DisGeNET databases, applying thresholds of a GeneCards score ≥ 0.05 and a DisGeNET score ≥ 0.1. Overlapping targets between RA-related genes and putative TFRD targets were identified through Venn diagram analysis using R software (version 4.5.2). A compound-target (C-T) interaction network was constructed using Cytoscape software (version 3.8.2), integrating active compounds and their corresponding and shared targets. Kyoto Encyclopedia of Genes and Genomes (KEGG) pathway enrichment analysis was performed to identify relevant signaling pathways, with pathways exhibiting *p*-values less than 0.05 considered statistically significant.

### 4.10. Western Blot Analysis

Total protein was isolated from ankle joint tissues or RAW264.7 cells using RIPA lysis buffer (P0013, Beyotime, Shanghai, China) and quantified via the BCA assay kit (P0010, Beyotime, Shanghai, China). Equivalent quantities of protein from each group were combined with loading buffer and subsequently subjected to denaturation at 100 °C. Following this, the protein was separated by sodium dodecyl sulfate–polyacrylamide gel electrophoresis (SDS-PAGE) and transferred onto a PVDF membrane (P2120, Millipore, Burlington, MA, USA). After blocking with 5% BSA, the membrane was incubated with anti-HIF-1α, HK2, PKM2, GLUT1 and GAPDH primary antibodies (Supplementary Table S1) overnight at 4 °C. The membrane was washed with TBST 3 times and then incubated with diluted secondary antibodies (1:10,000, SA00001, Proteintech, Wuhan, China) for 2 h at room temperature. Protein bands were visualized using ECL chemiluminescent reagent (PE0010, Servicebio, Wuhan, Hubei, China). The relative protein expression was quantified via Image J software (version 1.47), with GAPDH serving as the internal control.

### 4.11. Flow Cytometry Analysis

Flow cytometry analysis was conducted according to a previous study [62]. The joint tissues were minced and subjected to enzymatic digestion with collagenase II (17101015, Gibco, USA) at 37 °C for 1 h. The single-cell suspensions were obtained by passing the cells through a 70 μm cell filter (G6016, Servicebio, Wuhan, China). The single-cell suspensions from cultured RAW264.7 cells were harvested by utilizing a cell scraper. For macrophage polarization analysis, the FITC anti-CD86 and APC anti-CD206 antibodies were utilized to stain macrophages in PBS containing 5% (*v*/*v*) FBS at 4 °C. The CytoFLEX Sflow cytometry analyzer (Beckman Coulter, Indianapolis, IN, USA) was utilized to collect data.

### 4.12. Extracellular Acidification Rate Assays

The RAW264.7 cells were cultured in α-MEM medium containing 10% fetal bovine serum and 100 U/mL penicillin and streptomycin at 37 °C with 5% CO_2_. The extracellular acidification rate (ECAR) was assessed using the Seahorse XF Glycolysis Stress Test Kit (103020-100, Agilent, Santa Clara, CA, USA) according to the manufacturer’s protocol. In brief, RAW264.7 cells were plated onto 96-well XF cell culture microplates at a density of 1 × 10^4^ cells per well and incubated with or without 300 μg/mL of TFRD at 37 °C for 24 h. Simultaneously, the XFe96 FluxPak mini (102601-100, Agilent, Santa Clara, CA, USA) was immersed in Seahorse XF calibration solution (100840-000, Agilent, Santa Clara, CA, USA) and incubated overnight at 37 °C under the non-CO_2_ condition. Before testing, the culture medium was replaced with a XF glycolysis assay medium (103334-100, Agilent, Santa Clara, CA, USA) containing 2 mM/L of glutamine (103579-100, Agilent, Santa Clara, CA, USA). Then, the cells were incubated at 37 °C to equilibrate the extracellular environment. The data were acquired using a Seahorse XFe96 analyzer (Agilent, Santa Clara, CA, USA). Basal ECAR values were recorded under resting conditions. Subsequently, sequential injections of 10 mM glucose, 1 μM oligomycin, and 50 mM 2-deoxyglucose (2-DG) were administered to determine the basal glycolysis, maximal glycolytic capacity, and glycolytic reserve, respectively.

### 4.13. Glucose Uptake Assay

For the assessment of the glucose uptake, RAW264.7 cells were seeded into 24-well plates at a density of 1 × 105 cells per well and treated with 300 μg/mL of TFRD for 24 h. Before measurement, the culture medium was replaced with a serum-free growth medium containing 100 μM 2-(N-(7-nitrobenz-2-oxa-1,3-diazol-4-yl)amino)-2-deoxyglucose (2-NBDG) (HY-DY1019, MedChemExpress, Monmouth Junction, NJ, USA), and cells were incubated for 30 min at 37 °C in the dark condition. Following incubation, the supernatant was discarded, and cells were washed with PBS. Subsequently, flow cytometry was conducted to quantify the mean fluorescence intensity of 2-NBDG in cells from each group.

### 4.14. Enzyme-Linked Immunosorbent Assay

After stimulation, the supernatants were collected from RAW 264.7 cells and centrifuged to remove cellular debris. The serum was prepared by centrifugation of whole-blood samples at 4 °C for 15 min. The concentrations of IL-1β, TNF-α and IL-6 in the culture medium supernatants and mouse serum were quantified using commercial ELISA kits (Thermo Fisher, Waltham, MA, USA) according to the manufacturer’s instructions.

### 4.15. Overexpression of HIF-1α

Transient overexpression of HIF-1α in macrophages was achieved by using pcDNA3.1-overexpression plasmid and Lipofectamine 3000 transfection reagent (Tsingke Biotechnology, Beijing, China). In brief, RAW264.7 cells were seeded at 8 × 10^5^ cells/well and grown to 70–75% confluence in 6-well plates. An amount of 2.5 μg of pcDNA3.1- HIF-1α or empty plasmid per well was diluted in α-MEM, mixed with diluted Lipofectamine 3000, and incubated at room temperature for 15 min. The transfection complex was gently added to the cell culture medium. After 6 h of incubation, the medium was replaced with a complete medium. Cells were cultured for 24 h to obtain maximal gene overexpression before subsequent treatments and were then treated with LPS (100 ng/mL) in the presence or absence of TFRD for another 24 h. All cell samples were harvested within 48 h post-transfection. The cells transfected with empty pcDNA3.1 vector were established as the blank and negative control groups for the subsequent detection of HIF-1α expression, glycolytic activity, and macrophage polarization phenotypes.

### 4.16. 16S rRNA Analysis

The fresh fecal contents were collected from indicated mice, and the total bacterial DNA was extracted by using the DNeasy PowerSoil Pro Kit (Qiagen Sciences, Germantown, MD, USA) in accordance with the manufacturer’s protocol. The DNA concentration and purity were measured using a NanoDrop spectrophotometer, and the integrity was verified by 2% agarose gel electrophoresis. The DNA was amplified and sequenced by the Sanger sequencing method by GENEWIZ Co., Ltd. (Suzhou, China). In brief, the V3–V5 hypervariable region of the target DNA was employed by using a generic 16S rRNA gene primer [63]. Purified amplicons were subjected to library construction and sequenced on an Illumina MiSeq platform. Raw sequencing data were processed through the DADA2 pipeline for quality filtering, denoising, chimera removal, and amplicon sequence variant (ASV) clustering. Sequences containing ambiguous bases, shorter than 200 bp or with a Phred quality less than 20 were discarded, and chimeras were eliminated to obtain clean reads. The effective sequencing depth ranged from 76,190 to 101,283 reads per sample. OTU clustering was performed at 97% similarity using QIIME 1.9.1, and taxonomic assignment was conducted against the Silva_138 database. Alpha-diversity indices (Chao1, Shannon) were calculated, with intergroup comparisons performed by Kruskal–Wallis test. Principal component analysis (PCA) and principal coordinate analysis (PCoA) were utilized to assess the similarity of microbial community structures among samples. Additionally, the relative species abundance was examined at both the phylum and genus levels, and stacked bar charts were generated to illustrate the species composition.

### 4.17. Statistical Analysis

Statistical analyses were conducted using Student’s *t*-test for comparisons between two groups, while one-way or two-way ANOVA followed by Tukey’s multiple-comparisons test was employed for comparisons involving more than two groups. Non-normally distributed datasets were analyzed with the Kruskal–Wallis test followed by Dunn’s post hoc test. Longitudinal repeated measurements of arthritis scores and paw thickness were analyzed by repeated-measures one-way ANOVA with Tukey’s post hoc test to account for serial observations within individual animals. For gut microbiome differential-taxon analysis, the Benjamini–Hochberg false discovery rate (FDR) correction was applied for multiple-testing adjustment. A *p*-value of less than 0.05 was considered indicative of statistical significance. All analyses were performed using GraphPad Prism version 8.0 (GraphPad Software, Inc., La Jolla, CA, USA). Data are presented as means ± standard deviations (SDs).

## 5. Conclusions

In summary, our study demonstrated that TFRD alleviated inflammation and bone resorption in CIA mice partly by suppressing macrophage pro-inflammatory polarization. Mechanistically, the downregulation of HIF-1α-mediated glycolysis partially contributes to the inhibitory effect of TFRD on macrophage pro-inflammatory polarization. Furthermore, TFRD partially restored gut microbiota homeostasis and increased the abundance of Parabacteroides, suggesting that gut microbiota alterations may be associated with the beneficial effects of TFRD in RA treatment. Collectively, our findings provide preclinical evidence supporting the further investigation of TFRD as a potential therapeutic candidate for RA. Further studies should confirm the causal relationship between the TFRD-mediated inhibition of the HIF-1α pathway and the reduction of pro-inflammatory macrophages in RA.

## Data Availability

The original data presented in this study are included in the article/Supplementary Materials. Further inquiries can be directed to the corresponding authors.

## Supplementary Materials

The following supporting information can be downloaded at https://www.mdpi.com/article/10.3390/ph19101592/s1, Table S1: Primer sequences for RT-PCR. Table S2: Antibody list. Figure S1: UPLC-MS chromatograms of TFRD power. Figure S2: The MS/MS spectrum of naringin and neoeriocitrin. Figure S3: TFRD did not directly modulate osteoclast differentiation of BMDMs in vitro. Figure S4: Network pharmacology analysis of TFRD and RA.

## Abbreviations

The following abbreviations are used in this manuscript:

TFRD	Total flavonoids of Rhizoma Drynariae
RA	Rheumatoid arthritis
CIA	Collagen-induced arthritis
HIF-1α	Hypoxia-inducible factor-1α
BV/TV	Bone volume fraction
Tb.N	Trabecular number
Tb.Th	Trabecular thickness
Tb.Sp	Trabecular separation
BMDMs	Bone marrow-derived macrophages
