# Supplementary material for: Total Flavonoids of Rhizoma Drynariae Ameliorate Rheumatoid Arthritis by Modulating Macrophage Polarization and HIF-1α-Associated Glycolysis
Source: Pharmaceuticals (Basel). 2026 Oct 8;19(10):1592. doi: 10.3390/ph19101592 (PMC13708341; doi:10.3390/ph19101592)
Supplement: Supplementary file 1 [file pharmaceuticals-19-01592-s001.zip › pharmaceuticals-4589311-supplementary.pdf]

Supplementary Table S1. Primer sequences for RT-PCR.

Primer name	Sequences (5'-3')
TRAP_F	GGACAGGACACACACACACA
TRAP_R	CAAACAGGAGAGCCACTTCA
CTSK_F	TCCCTGAACTCTGCACCAAG
CTSK_R	ATCTGGCTCAGGTAGGAGGG
Nlrp3_F	CCTTGGTGAGGTTTGATCCGCATA
Nlrp3_R	GCACCCATGGCAGAAGGAGG
Hif-1α_F	TCAAGTCAGCAACGTGGAAG
Hif-1α_R	TATCGAGGCTGTGTCGACTG
GAPDH_1 F	GAGAAACGGCTACCACATCC
GAPDH_1 R	CCTCCAATGGATCCTCGTTA

Supplementary Table S2. Antibody list

1st antibody	Company	Catalogue Number	Application	Dilution
HIF-1α	Abcam	ab179483	WB/IF/ICC	1:1000/1:200/1:500
CD86	Abcam	ab239075	IF/ICC	1:200/1:500
CD206	Abcam	Ab8918	ICC	1:500
F4/80	Abcam	ab6640	IF	1:200
HK2	Proteintech	22029-1-AP	WB	1:1000
PKM2	Proteintech	15822-1-AP	WB	1:1000
GLUT1	Proteintech	21829-1-AP	WB	1:1000
APC anti-CD206	BioLegend	A17199A	FACS	1:100
FITC anti-CD86	BioLegend	C068C2	FACS	1:100
GAPDH	Proteintech	10494-1-AP	WB	1:1000

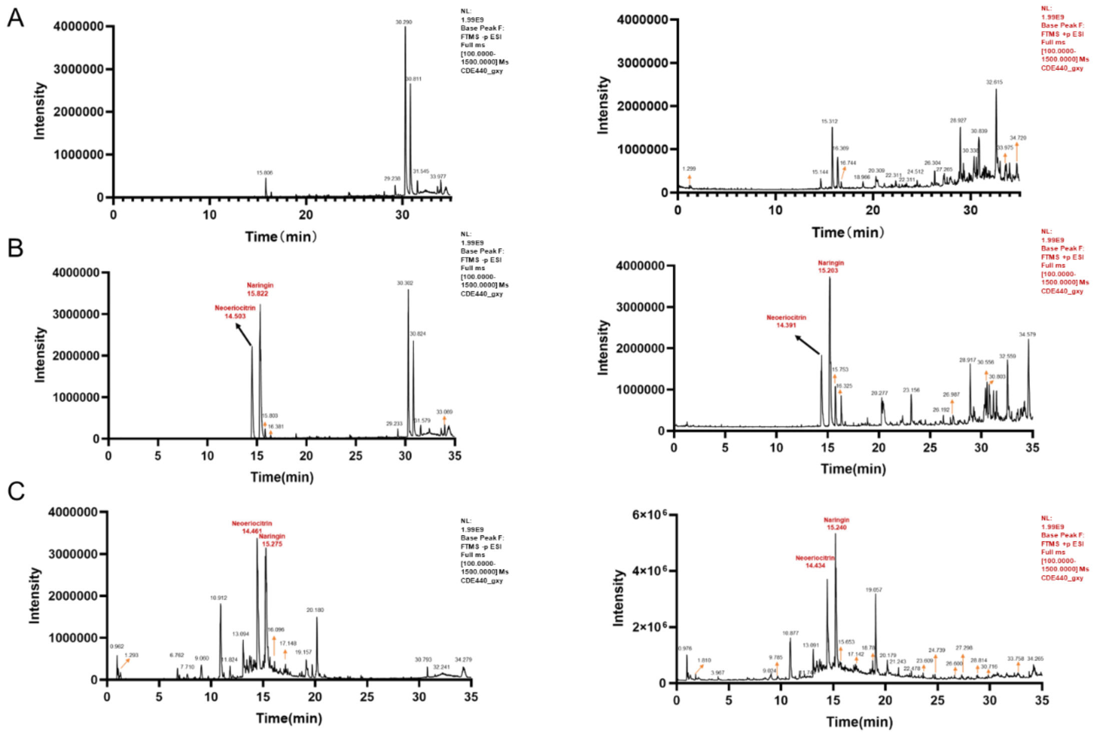

Supplementary Figure S1. UPLC-MS chromatograms of TFRD power. Base-peak chromatograms of (A) Blank, (B) standard sample containing naringin and neoeriocitrin, and (C) TFRD power in (left) negative -ion and (right) positive-ion ESI modes. The naringin and neoeriocitrin has been assigned.

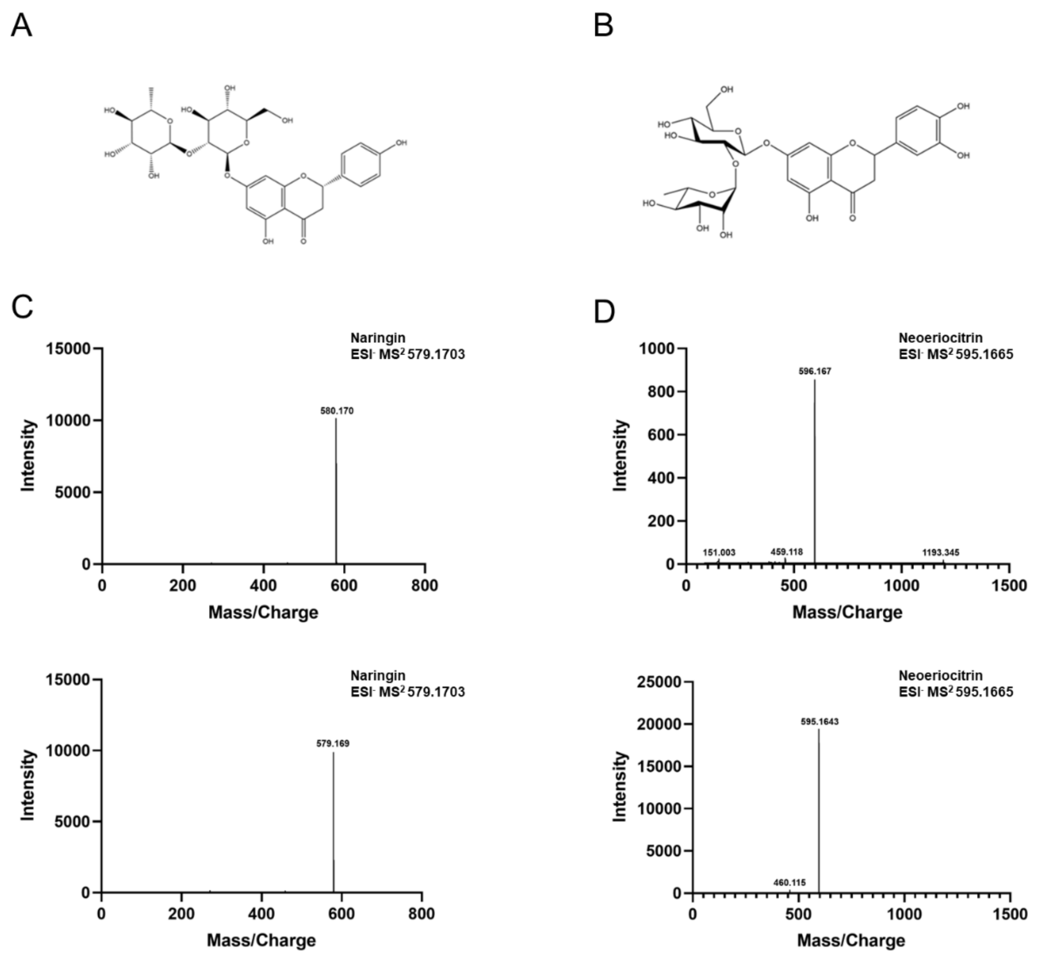

Supplementary Figure S2. The MS/MS spectrum of naringin and neoeriocitrin. The structural formula of (A) naringin (B) and neoeriocitrin. (C) The secondary mass spectrum of naringin in (up) standard sample and (down)TFRD sample. (D) The secondary mass spectrum of naringin in (up) standard sample and (down)TFRD sample.

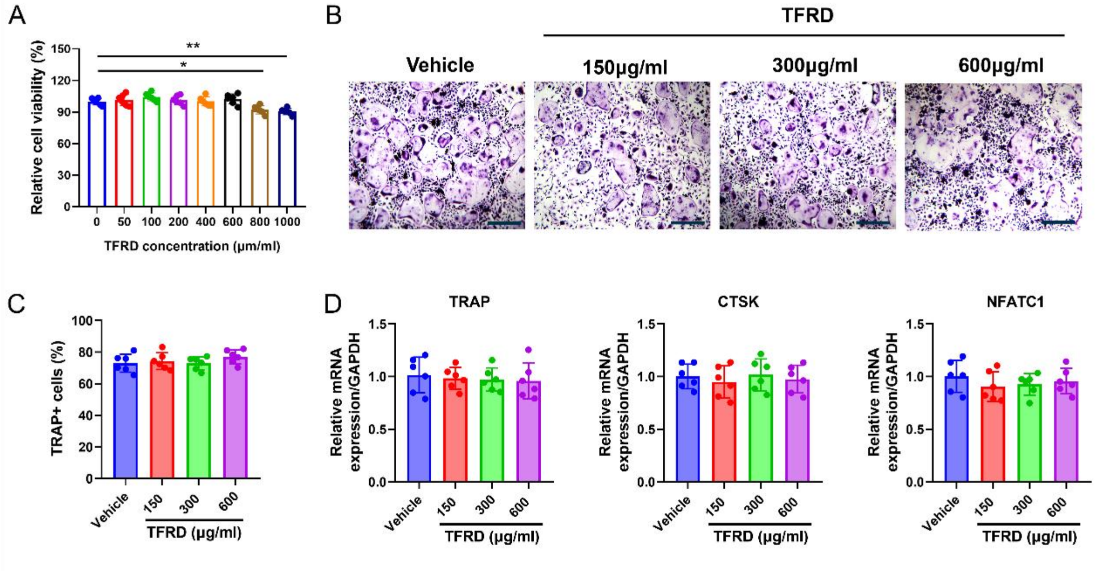

Supplementary Figure S3. TFRD did not directly modulate osteoclast differentiation of BMDMs in vitro**.** (A) The effect of TFRD gradient on cell viability of BMDMs. (B) Representative images of TRAP staining of BMDMS after 5-days induction with RANKL and TFRD, Scale bar = 200 μm. (C) Quantitative analysis of TRAP+ cells from B. (D) Quantitative analysis of the gene expression of TRAP, CTSK and NFATC1. Data are presented as the mean ± SD, *N*=6 for each group, *: *P*<0.05; **:*P*<0.01.

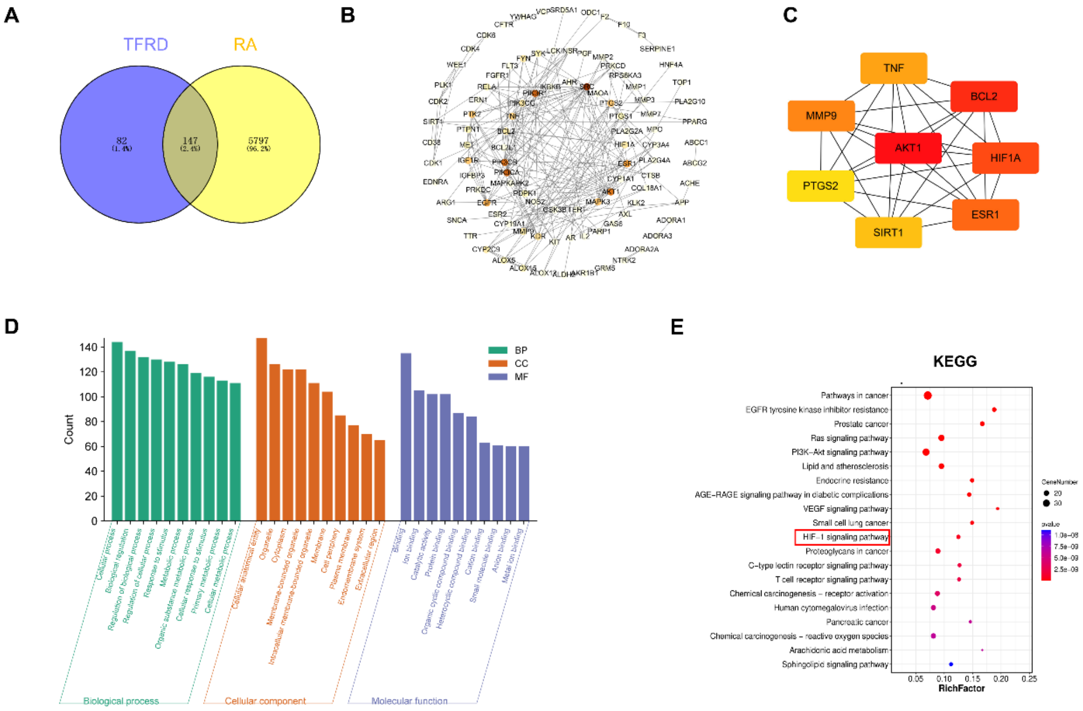

Supplementary Figure S4. Network pharmacology analysis of TFRD and RA. (A) Venn diagram showing 147 overlapping targets between TFRD and RA. (B) The PPI network. (C) Top 8 targets based on degree values. (D) GO enrichment analysis of intersecting drug-disease genes, showing the top 20 terms across on biological process (BP), cell components (CC) and molecular functions (MF). (E) KEGG pathway enrichment analysis of the top 20 pathways.
